# Lack of association between VEGF -2578C/A polymorphism and risk of colorectal cancer in an Iranian population 

**Published:** 2020

**Authors:** Sanaz Savabkar, Neda Zali, Mahrooyeh Hadizadeh, Shabnam Tavangarroosta, Chris Young, Fateme Shojaeian, Nastaran Ebrahimi, Maziar Ashrafian Bonab, Hamid Rezvani, Farzaneh Shalileh, Ehsan Nazemalhosseini-Mojarad

**Affiliations:** 1 *Basic and Molecular Epidemiology of Gastrointestinal Disorders Research Centre, Research Institute for Gastroenterology and Liver Diseases, Shahid Beheshti University of Medical Sciences, Tehran, Iran *; 2 *School of Medicine, University of Sunderland, City Campus, Chester Road, Sunderland, SR1 3SD, UK*; 3 *School of Allied Health Sciences, Faculty of Health and Life Sciences, De Montfort University, Leicester, LE1 9BH, UK *; 4 *Gastroenterology and Liver Diseases Research center, Research Institute for Gastroenterology and Liver Diseases, Shahid Beheshti University of Medical Sciences, Tehran, Iran*

**Keywords:** Colorectal cancer, Angiogenesis, VEGF, Single nucleotide polymorphism, Vascular endothelial growth factor

## Abstract

**Aim:**

Here, we evaluated the VEGF gene -2578C/A polymorphism as a potential susceptibility factor in colorectal cancer (CRC) occurrence amongst Iranian CRC patients.

**Background:**

Vascular endothelial growth factor (VEGF) is a key regulatory factor in angiogenesis which plays essential roles in the development of malignancy in colorectal cancer (CRC), as the third most prevalent cancer worldwide.

**Methods:**

VEGF -2578C/A polymorphism was evaluated in 200 CRC patients and 200 healthy control subjects via restriction fragment length polymorphism analysis.

**Results:**

The frequencies of CC, AC and AA genotypes among CRC patients were 22.5%, 51% and 26.5%, respectively, with their respective genotype frequencies at 16%, 54% and 30% in control cohorts (P=0.247). The A allele frequency among the case group was 52% and for control group, it was 57%. C allele frequency in case and control groups was 48% and 43%, respectively (p=0.156). No significant association was observed (p=0.990) between this polymorphism and CRC stage.

**Conclusion:**

Our findings

provide limited support for the hypothesis that the -2578C/A VEGF are associated with increased risk of colorectal cancer in Iranian colorectal cancer patients and suggest instead that meta data studies, which have previously relied upon populations definitions such as ‘Asian’, should more specifically take into account country of origin when associating prognostic value to a given genotype.

## Introduction

 Colorectal cancer is the third most prevalent type of cancer word-wide (1-5) and is becoming a major health concern in Asia (6-8). There have been several studies evaluating the association of single nucleotide polymorphisms in cancer-related genes and CRC susceptibility (9-14). Clinical studies have previously shown that the dominant angiogenesis factor in colorectal cancer is VEGF through mechanism such as promoting angiogenesis and the reproduction and survival of endothelial cells (15-17). VEGFA is known generally as VEGF (17,18). VEGF gene has been mapped to chromosome 6p12-p21 and is comprised of 8 exons and 7 introns (19,20). Increased VEGF levels have previously been reported in tumour cells, and the intensity of small vessels around tumours were directly related to VEGF levels (21,22). Moreover, it has been reported that VEGFA is overexpressed in 50% of colorectal cancers. 

Functional studies have shown that VEGF polymorphisms can modify cancer susceptibility (23-25); - 2578A > C is associated with a higher VEGF expression, for example (26). Koukourakis et al. (23) reported the −2578C/C genotype correlated with reduced VEGF expression, while the −2578C/A was associated with higher VEGF levels in non-small cell lung cancers. Single nucleotide polymorphisms (SNPs) are a significant source of genomic heterogeneity, which may alter cancer susceptibility (27,28). Several SNPs have been identified in VEGF gene including -2578C/A (rs699947), which is located on promoter region of VEGF and is known to alter VEGF protein expression (23,29-31). This putative polymorphism is shown to be associated with diabetic, CRC and breast cancer (29,32,33). The association of the SNPs of VEGF with clinical outcome of patients with oesophageal cancer, lung cancer and gastric cancer was shown previously in public data sets (23,34, 35).

We hypothesized that these polymorphisms could also have an impact on CRC susceptibility. Therefore, the aim of the present study was to investigate whether -2578C/A polymorphism in the VEGF gene is involved in the development of sporadic colorectal cancer in Iranian patients, and to evaluate its usefulness as a prognostic marker. 

## Methods

**Study population**

Peripheral blood samples investigated in this study were obtained from 200 CRC patients and 200 healthy subjects, between 2009 and 2011, who had been referred to the Research Centre for Gastroenterology and Liver Diseases (RCGLD), Shahid Beheshti University of Medical Sciences (Tehran, Iran). Written informed consent for this study was received from patients and ethics approval was granted by the ethics committee of the Gastroenterology and Liver Diseases Research Centre, Shahid Beheshti University of Medical Sciences, Tehran, Iran.

All patients were histologically diagnosed as being positive for colorectal cancer at the RCGLD. The control group included individuals who referred to this centre for screening purposes and whose negative colonoscopy procedure approved that they are not susceptible to CRC. The parameters of age, gender and cigarette smoking status were collected from patients and control groups. The stage of CRC was also determined in the patient group. The diagnosis and staging of colorectal cancer were assessed according to the WHO classifications after confirmation by accredited pathologists. The cases were then submitted for genetic studies.

**Genotyping**

Total genomic DNA was extracted from peripheral blood using the Salting Out method (36). Primer sequences of the VEGF gene were designed with Gene Runner software (version 4.0.9.68 Beta). The PCR primers used to amplify the promoter region of VEGF gene included the following: Forward: 5'- ACTAGTGCACGAATGATGG-3' and the Reverse primer: 5'- ATTCCTAGCTGGTTTCTGAC -3'. Synthesis of the suitable size (385 bp) PCR products was confirmed by agarose gel electrophoresis. Genotyping was performed by polymerase chain reaction–restriction fragment length polymorphism (PCR-RFLP). The VEGF (-2578C/A) PCR product was digested with restriction endonuclease BglII (sequence of restriction site: AGATCT) for 5h at 37°C. The PCR fragments of the A/C genotype were digested into fragments of 113,272bp and 385bp. AA genotype was also digested into two fragments of 113bp and 272 bp and CC genotype was digested into a 385-bp product. Fragments were analysed on 3% agarose electrophoresis gels visualised using a UV-based trans-illuminator to detect ethidium bromide staining.

**Statistical analysis**

Unconditional logistic regression analysis was conducted to estimate adjusted and unadjusted odds ratio (OR) and 95% confidence interval (CI) to determine association of -2578C/A with the risk of CRC. OR and 95% CI were adjusted for age, sex and smoking status. In CRC patients, the coloration between the clinical pathology features and polymorphism were examined using chi-square test. Data were considered significant when the statistical p-value was <0.05. All the statistical analyses were carried out using SPSS (v.13). 

## Results

VEGF genotypes did not deviate from the Hardy–Weinberg equilibrium among patients or controls (p=0.759, p =0.151, respectively for case and controls). Table 1 shows the genotype and allelic distributions of the -2578C/A polymorphism in cases vs. controls. We found no statistically significant association between this polymorphism and the risk of colorectal cancer (p=0.156, OR=1.224, CI=0.926–1.617). Characteristics of patients and control subjects including age, smoking habit and gender are summarized in Table1.

 No significant differences were observed in age or smoking between the two groups. Correlation between the stage of the disease and genotype was shown in Table 2. The genotyping frequency of -2578C/A polymorphism was not associated with tumor stage (p=0.999).

**Table 1:** Characteristics of populations

Variables	Cases (N=200)	Control (N=200)	^1^ *P value*
Age (mean ± SD)	58.37±12.998	45.27±13.151	
Gender Female 95 (47.5%) 115 (57.5%) Male 105 (52.5%) 85 (42.5%)			
Smoking Never 182 (91.0%) 173 (86.5%) 0.154 Ever 18 (9.0%) 27 (13.5%)			
Genotype CC AC AA	45 (22.5%) 102 (51.0%) 53 (26.5%)	32 (16.0%) 108 (54.0%) 60 (30.0%)	0.247
Allele A C	208 (52.0%) 192 (48.0%)	228 (57.0%) 172 (43.0%)	0.156

^1^ Significant change (P value < 0.05) when compared to the control.

**Table 2:** The tumour-stage shows specific distribution of VEGF -2578C/A genotypes among colorectal cancer patients

Genotype	Stage 0	Stage I	Stage II	Stage III	Stage IV	*P* value
CC	1 (10.0%)	6 (22.2%)	11 (22.0%)	16 (22.9%)	11 (25.6%)	
AC	5 (50.0%)	14 (51.9%)	26 (52.0%)	36 (51.4%)	21 (48.8%)	0.990
AA	4 (40.0%)	7 (25.9%)	13 (26.0%)	18 (25.7%)	11 (25.6%)	

## Discussion

VEGF binding to VEGFR leads to the activation of many signalling pathways such as Ras/Raf/MAPK which are involved in cell growth, cell proliferation, angiogenesis and survival of endothelial cells (37). VEGF is a master mediator of vascular permeability with a significant role in angiogenesis and metastasis of human cancer including colorectal cancer (38). VEGF gene is highly polymorphic and at least 30 single-nucleotide polymorphisms (SNP) in this gene have been described in the literature (39-42). Functional polymorphisms may influence the expression of a gene (29). This effect may be due to only one polymorphism, or a combined effect of more than one polymorphism (29). It has been shown that polymorphisms located in promoter, 5′ and 3′ untranslated regions of the VEGF gene have functional influence on VEGF gene expression (43-46). In the present study, the VEGF -2578C/A polymorphism did not alter the risk of developing colorectal cancer in our Iranian population (p=0.247). However, Park et al. reported a different frequency of this genotype in a Korean population (32). Similar to our results, Hoffmann et al. have shown no association of VEGF gene-2578C/A polymorphism with the risk of colorectal cancer in a population (p=0.23) (38). Also, in line with our study, results of Vidurrent et al. showed a similar distribution (47,48). In contrast, Howell et al. and Yang et al. found a significant association between -2578C/A polymorphism and the risk of breast cancer and malignant melanoma in Caucasians (29,49). This conflicting result might be due to several reasons including ethnic background, genotype distributions, environment factor and other clinical factors (50).

Koukourakis et al. reported the correlation between -2578CC genotype and low expression of VEGF, while -2578C/A genotype was correlated with an increase in the expression of VEGF in lung tumour cells (23). Also, Tzanakis et al. reported a significant correlation between -2578AA and size of tumour (P=0.025), as well as low distinction and progress of gastric cancer in a Greek population (P=0.039) (31). In the present study, we found no significant differences between the cases and controls for the demographic data (age, gender and smoking). Moreover, our results confirmed observations by Hoffmann et al. (34) and Dassoula et al. (51) on demographic features in colorectal cancer risk. However, Park et al. reported that the frequency of the –2578CA+AA genotype in patients was associated with reduced risk for colon cancer in women (OR, 0.60; 95% CI, 0.36-0.99; p=0.056). Also, frequency of the -2578CA+AA genotype was protective against colon cancer in patients with proximal colon cancer (OR, 0.55; 95% CI, 0.31-0.97; p=0.049). Therefore, the effects of VEGF genotype may be different in the two genders. There was no difference when the data was stratified according to gender. According to the previous study by Dassoula et al. (51), tumour phenotype can be related to the specific organ of origin. Each organ has its own individual genotype and phenotype profile. The present study had some limitations. The patients were recruited from a single centre. Also, no other SNPs in this gene were evaluated and associations of other genes in angiogenesis with VEGF were not explored. In conclusion, we did not find that VEGF -2578C > A polymorphism was associated with the risk of CRC in Iranian population. Nevertheless, further studies will be needed to explore the complicated interaction between environmental factors and VEGF -2578C>A polymorphism in susceptibility to CRC. This study highlights the significant need to take into account specific country of origin during SNP analyses. Meta-data analyses using grouped classifications such as ‘Asian’ are inappropriate due to regional variations in SNPs.
